# Correction: Canadian adolescents’ perceptions of how climate change is impacting their mental health: A qualitative analysis of open-ended survey responses

**DOI:** 10.1371/journal.pmen.0000520

**Published:** 2025-12-17

**Authors:** Gina Martin, Ishwar Tiwari, Rebekah Alice McKinnon, Azeezah Jafry, Ekroop Grewal, Jason Gilliland, Kendra Nelson Ferguson, Kiffer G. Card, Maya Gislason, Alina Paula Cosma

In Table 1, there is a typographical error in the “Climate change is impacting mental health” cell.

The word “Unknown” should read as “No”. Please see the correct Table 1 here.

**Table 1:** Socio-demographic characteristics of the overall participants (N = 804) and of the subset of study participants whose open-ended responses were analyzed (N = 235).

Sample characteristic	Full Sample	Subset with Open-ended Responses
	(n = 804)	(n = 235)
	n (%)/ Mean (SD), Minimum, Maximum	n (%)/ Mean (SD), Minimum, Maximum
**Age**	15.6 ((± 1.65), 13, 18	15.7 (± 1.71), 13, 18
**Province**		
Alberta	123 (15.0%)	42 (17.9%)
British Columbia	123 (15.0%)	35 (14.9%)
Manitoba	70 (8.7%)	19 (8.1%)
New Brunswick	48 (6.0%)	15 (6.4%)
Newfoundland and Labrador	26 (3.2%)	8 (3.4%)
Northern Territories*	28 (3.5%)	6 (2.5%)
Nova Scotia	52 (6.5%)	13 (5.5%)
Ontario	135 (17.0%)	41 (17.4%)
Prince Edward Island	2 (0.2%)	-
Quebec	128 (16.0%)	35 (14.9%)
Saskatchewan	69 (8.6%)	21 (8.9%)
**Urban/rurality**		
City	550 (68.4%)	160 (68.1%)
Rural or remote area	65 (8.1%)	20 (8.5%)
Small town	187 (23.3%)	54 (23.0%)
Missing	2 (0.2%)	1 (0.4%)
**Gender**		
Girl	407 (50.6%)	129 (54.9%)
Boy	371 (46.1%)	98 (41.7%)
Gender diverse	21 (2.6%)	8 (3.4%)
Prefer not to answer/ Another identity not listed	5 (0.6%)	-
**Race/ethnicity**		
White	458 (57.0%)	144 (61.3%)
Black	96 (11.9%)	26 (11.1%)
South Asian	45 (5.6%)	16 (6.8%)
Indigenous (First Nation, Inuit/Inuk, Metis)	11 (1.4%)	5 (2.1%)
Other race or ethnicity (including multiple)	177 (22.0%)	42 (17.9%)
Prefer not to answer	17 (2.1%)	2 (0.9%)
**Perceived Family Affluence**		
Not well-off	118 (14.6%)	49 (20.9%)
Average	385 (48.0%)	110 (46.8%)
Well-off	299 (37.2%)	76 (32.3%)
Missing	2 (0.2%)	-
**Climate change is impacting mental health**		
No	493 (61.3%)	-
Yes, a little	212 (26.4%)	178 (75.7%)
Yes, a lot	79 (9.8%)	57 (24.3%)
Not asked/ Missing	20 (2.5%)	-

SD: Standard Deviation

* Northern Territories: includes Northwest Territories, Yukon, and Nunavut.
