# Supplementary material for: Canadian adolescents’ perceptions of how climate change is impacting their mental health: A qualitative analysis of open-ended survey responses
Source: PLOS Ment Health. 2025 Sep 24;2(9):e0000424. doi: 10.1371/journal.pmen.0000424 (PMC12798330; doi:10.1371/journal.pmen.0000424)
Supplement: Attachment — Submitted filename: Response to Reviewers comments.docx [file pmen.0000424.s004.docx]

July 28, 2025

Paolo Raile

Academic Editor

PLOS Mental Health

Dear Editor,

We sincerely appreciate the time and effort invested by you and the reviewers in evaluating our manuscript. The constructive feedback provided has been invaluable in enhancing the quality, clarity, and rigor of our work.

Below, we have carefully addressed each of the reviewers’ comments in a point-by-point manner and incorporated the suggested revisions into the manuscript. We hope that our responses and modifications adequately address all concerns raised and further strengthen the paper.

Thank you once again for your guidance throughout this process. We look forward to hearing about the next steps regarding our submission.

Thanks,

Dr. Martin

**Editor’s comment**

**1. Please ensure that your Ethics Statement is available in its entirety at the beginning of your Methods section, under a subheading 'Ethics Statement'.**

We added Ethics Statement at the beginning of the Methds section (Section: Methods, 2.1 Ethics Statement). We stated:

“The study procedures were approved by the Athabasca University Research Ethics Board (#25013). Before undertaking the survey, potential participants were provided with a Letter of Information outlining the aims, length, and content of the survey. Potential participants were assured of the anonymity and confidentiality of their data, as well as the voluntary nature of their participation. We obtained written consent from the participants before they proceeded with the survey questions. Participants received compensation for their time, as per the terms they agreed to when joining the panel, for example, participants could elect to get points which they could redeem with specific merchants.”

**2. For studies involving third-party data, we encourage authors to share any data specific to their analyses that they can legally distribute. PLOS recognizes, however, that authors may be using third-party data they do not have the rights to share. When third-party data cannot be publicly shared, authors must provide all information necessary for interested researchers to apply to gain access to the data. (https://journals.plos.org/plosone/s/data-availability#loc-acceptable-data-access-restrictions) 
 For any third-party data that the authors cannot legally distribute, they should include the following information in their Data Availability Statement upon submission:
 1) A description of the data set and the third-party source
 2) If applicable, verification of permission to use the data set
 3) Confirmation of whether the authors received any special privileges in accessing the data that other researchers would not have
 4) All necessary contact information others would need to apply to gain access to the data**

We appreciate your suggestions. We have provided Data Availability Statement at the end but before the references. We stated:

“The dataset is not available for sharing publicly because we do not have ethical approval to share beyond our immediate research team. The Research Ethics Board who has imposed this restriction is Athabasca University Research Ethics Board (contact: rebsec@athabascau.ca). Anonymized data may be made available, upon reasonable request, to the corresponding author and following ethical approval.”

**Reviewer #1:**

Feedback on Manuscript: Canadian Adolescents' Perceptions of how Climate Change is Impacting their Mental Health: A Qualitative, Cross-sectional Analysis

**General Comments**:
This manuscript presents a well-conducted qualitative study exploring Canadian adolescents’ perceptions of how climate change impacts their mental health. The topic is timely and significant, given the growing recognition of climate change as a determinant of mental health, particularly among youth. The use of open-ended survey responses to capture nuanced perspectives is a strength, as is the involvement of youth co-researchers, which enhances the study’s authenticity and relevance. The thematic analysis is robust, and the findings align with existing theoretical frameworks while offering new insights into adolescents’ lived experiences. However, there are areas where clarity, methodological rigor, and discussion could be strengthened to enhance the manuscript’s impact. Below, I provide detailed feedback organized by section, followed by specific recommendations for improvement.
**Strengths**:
1. Relevance and Originality: The study addresses a critical gap in the literature by focusing on adolescents’ qualitative perspectives on climate change’s mental health impacts, an area underexplored compared to quantitative studies. The findings contribute to understanding the diverse pathways (direct, indirect, and overarching) through which climate change affects youth mental health.
2. Youth Involvement: The inclusion of youth co-researchers in survey design and coding is a significant strength, aligning with participatory research principles and ensuring the study reflects the voices of the target population.
3. Thematic Analysis: The inductive thematic analysis with semantic coding is well-executed, with clear descriptions of the coding process, including the use of multiple coders and consensus-building to ensure rigor. The four identified themes (emotional/psychological responses, concerns for the future, impacts on functioning, and concerns for the environment/humanity/wildlife) are well-supported with participant quotes.
4. Contextual Relevance: The study is grounded in the Canadian context, referencing specific climate-related events (e.g., wildfires, heatwaves) that resonate with participants’ experiences, making the findings relevant to policymakers and public health practitioners in Canada.
5. Ethical Considerations: The manuscript clearly outlines ethical approvals, participant consent processes, and data anonymity, adhering to high ethical standards.
**Overall Assessment:**
This manuscript makes a valuable contribution to the literature on climate change and adolescent mental health, particularly by amplifying youth voices through qualitative methods. The thematic analysis is rigorous, and the findings offer actionable insights for policymakers and public health practitioners. However, addressing the identified areas for improvement particularly clarifying the research gap, enhancing methodological transparency, and strengthening the discussion’s global context will elevate the manuscript’s impact and suitability for publication in PLOS Mental Health. I recommend major revisions to address these points, with a focus on streamlining the manuscript and enhancing its theoretical and methodological clarity.

**Specific Recommendations:**
1. Introduction:
o **Add a paragraph or sentence comparing this study to existing qualitative work globally to clarify its unique contribution.**

Thank you for your comment. We have now added a sentence with appropriate citation comparing this study to existing global qualitative work. This addition appears at the end of the second-last paragraph and highlights a lack of such research in Canada.

o **Explicitly link the theoretical framework (direct, indirect, overarching pathways) to the study’s research questions and analysis plan.**

We have now a sentence at the end of the last paragraph that explicitly linking the theoretical framework to the study’s research questions and analysis plan.

2. Methods:
**o Provide more detail on the Qualtrics panel recruitment process, including how participants were selected and any incentives offered beyond “compensation per panel terms.”**

We have provided more information on this in the methods section (Section 2.1, paragraph 2). Specifically, the participants were targeted based on characteristics which were recorded by Qualtrics when joining the research panel and screened through eligibility questions. They were compensated based on what they selected when joining the research panel – for example, points that can be redeemed for gift cards or with specific merchants.

o **Clarify the criteria for excluding vague or irrelevant responses, potentially including examples in the supplementary material.**

We apologize for the lack of clarity regarding exclusion criteria. We have rephrased the sentence to explicitly outline two key exclusion criteria (Section 2.4 Data Analysis, paragraph 1).

o **Discuss strategies to address the underrepresentation of Francophone and non-English-speaking communities in future research.**

We have added two strategies to improve inclusivity in future research. The first strategy includes consideration of both English and French language for broader inclusivity, whereas the second strategy emphasizes on in-person or telephone interview to reach those with limited internet access.

3. Results:
o **Justify the retention of low-frequency subthemes or consider merging them with broader categories to streamline the findings.**

Thank you for your suggestion. We have provided the following justification in text (section: Thematic Results, paragraph 2).

Some subthemes were less frequently coded; however, we retained them as standalone categories to uncover nuanced perspectives, thus maintaining consistency with our inductive, participant-focused approach.

o **Include additional participant context (e.g., region or urban/rural status) with quotes to reflect Canada’s geographic diversity.**

As suggested, we have added region and urban/rural status for all quotes.

**o Integrate code frequencies more explicitly in the narrative to highlight the relative prominence of themes.**

Code frequencies were included in the original submission. We have now reorganized this information and included percentage in text for greater clarity across the results section.

4. Discussion:
o **Expand the comparison with global studies to situate the findings in a broader context.**

We have now included a comparison with findings in a broader context citing a paper by Hickman et al. However, we note that this study does not address mental health impacts explicitly but rather worry and functional impacts, as to the best of our knowledge there isn’t published research that asks about perceived mental health impacts specifically we can cite:

“While asking about feeling worry rather than mental health impacts, Hickman et al. (28) conducted a global survey of 10,000 children and young people (aged 16-25 years) in ten countries, which revealed that 59% of the participants felt ‘very’ or ‘extremely’ worried about climate change, while 84% were at least moderately worried.”

o **Provide more specific recommendations for interventions tailored to the identified themes (e.g., addressing guilt about parenthood or systemic barriers).**

Thank you for your recommendation. We have added some intervention suggestions for the various subthemes but do so with caution given a lack of research on efficacy of such interventions. For example, promoting family emotion literacy in both households and schools could improve psychological wellbeing and foster a positive outlook on the future, shaping fertility intentions. We have also explicitly mentioned recommendations to address systemic barriers. (Section: Implication, paragraph 2).

“Youth engagement in climate change discussions and decision-making processes can empower young people while addressing the systemic barriers and their concerns about climate change.”

o **Acknowledge the potential influence of recent climate events (e.g., 2023 wildfires) on participants’ responses and discuss how longitudinal studies could address temporal variations.**

We have added this to the limitations section:

“Canada’s 2023 wildfire season was the most destructive ever recorded at the time, which consumed 15 million hectares of land (62) preceding the timing of this survey, resulting in its potential influence on participants’ responses.”

We also stated:

“Future longitudinal studies could clarify the association between direct exposure to such extreme events and mental health impacts, addressing the temporal variations.”

5. Clarity and Presentation:
o **Define key terms (e.g., climate anxiety, climate distress) early in the manuscript and use them consistently.**

Since these terms were not used widely throughout the manuscript and because there are varying definitions used throughout the literature, we have removed them to avoid confusion.
o **Verify the accuracy of Table 1 percentages and ensure consistency across the text and tables.**

Thank you for pointing it out. We have now verified and ensured consistency across the text and tables.

o Condense the manuscript by summarizing repetitive quotes or sections to reduce the word count to approximately 5000–5500 words for better readability.

We appreciate this feedback. However, after addressing all reviewers’ comments, the manuscript exceeds the words limit suggested by the reviewer. To reduce the words, we would need to remove several quotes or move quotes into standalone tables – which we think will greatly reduce the flow of the results section which was written to narratively integrate the quotes; additionally, we think providing a couple quotes better illustrate the themes.

6. Supplementary Material:
o **The supplementary material is referenced but not described in detail. Provide a brief overview of its contents (e.g., list of 85 subcodes) in the main text or ensure it is accessible for reviewers to assess its relevance.**

We have added:

“The coding process identified 85 subcodes which were grouped into 42 parent codes provided as supplementary material.”

**Reviewer #2:**

This article, “Canadian Adolescents’ Perceptions of how Climate Change is Impacting their Mental Health: A Qualitative, Cross-sectional Analysis”, makes a timely and meaningful contribution to the intersection of adolescent mental health and climate science. The use of inductive thematic analysis on open-ended responses offers rare and valuable insights into how Canadian adolescents personally experience climate-related emotional burdens. The authors have done commendable work in elevating youth voices and presenting a nuanced picture of emotional, existential, and functional impacts.

**Strengths**:
The methodological design is robust, with clear justification for qualitative approaches and quota-based sampling ensuring geographic, gender, and age diversity.

The involvement of youth co-researchers throughout the process enhances the study’s credibility and relevance.

The five subthemes within emotional and psychological responses are particularly well-synthesized and illustrative.

**Recommendations**

**Clarify demographic limitations: While the paper mentions quota fulfillment, it would benefit from more detail on how representative the sample is of racialized youth, those from Francophone and Indigenous communities, and adolescents without internet access.**

We have clarified the sample’s characteristics and highlight this in contrast to some details from the 2021 census, although data are not available for 13-18 but rather 15-19 from the census, for racialized youth and Indigenous identity. National data on adolescents without internet access were unavailable but we note that this limitation likely led to underrepresentation of those living in remote communities. We also include an estimate from the census of those who use only French language

**Deepen intersectional analysis: Some respondents’ quotes suggest layered vulnerabilities (e.g. gender-diverse youth, those with pre-existing mental health conditions), but this nuance could be explored further in discussion.**

Only a few participants who provided statements were gender-diverse (n =8) or reported pre-existing mental health conditions in the quotes, limiting extensive intersectional analysis. However, we have added to implication section, paragraph 1, to highlight that this is an area for future research:

“Additionally, responses from gender-diverse participants and those with existing mental health conditions revealed that there might be some intersecting factors that could put some people at greater risk for the mental health impacts of climate change, which warrant further exploration.”

**Actionable implications: The “Implications” section could be expanded to include more specific, youth-informed policy recommendations or examples of promising practices (e.g. school-based supports, community resilience programs).**
We have included specific recommendations (Implication, paragraph 2) that might be promising, however, noting that we need more evaluation for such programs to determine efficacy and to avoid any unintended harms:

Further, school-based programs that offer young people opportunities to develop a sense of agency and empowerment about climate change in a supportive environment may support adolescents’ mental wellbeing.

Emotional literacy could be encouraged within families and groups that work with young people to support psychological wellbeing when dealing with uncertain outlooks toward the future; this may also create an outlet for sharing and discussing concerns around parenthood.

Public health organizations should also acknowledge and plan for the mental health challenges to adolescents that stem from climate change. Additionally, school and community design that aims to mitigate the impact of climate-related events on health, such as shaded outdoor spaces, is an important area for future work.

**Thematic contextualization: A table cross-referencing themes with demographic factors (e.g. age or region) could add further depth and help readers understand if different impacts are more common among certain subgroups.**
We have added participants age, gender, area of residence, and province for each quote included in the results section, which adds greater depth and help readers contextualize the impacts of climate change among different subgroups.

**Reviewer #3:**

The article used open-ended responses to a question in a cross-sectional survey to assess how climate change was impacting Canadian’s adolescents.

The article addressed an important question. Collection of primary data, specifically, a large nationally representative sample, from Qualtrics is a strength. The analyses are thorough, with the involvement of youth co-researcher a particular strength. The findings are informative. The paper is generally well written, giving voices to a largely neglected yet very important population in this sphere.

Below are my minor suggestions to further improve the paper:

**1) Qualitative Methodology: Perhaps it is my own limitation, but cross-sectional survey is usually used to describe quantitative research design. Qualitative research usually includes methods like interviews or field observations. The combination of “qualitative, cross-sectional” comes across a bit odd or awkward. Perhaps you can simply refer to it as an open-ended survey.
In addition, it would be helpful to clarify your epistemological stance, whether it is positivist or interpretivist.**

Thank you. We have revised the title as suggested and feel this is a good reflection of the paper; we have also clarified our epistemological stance (post-positivist) (Section: data analysis, paragraph 2).

**2) Participant: Please clarify, at the very beginning of the Methods section, what is your population of interest. Inclusion and exclusion criteria should be listed before description of any procedures.**

We clarify that our eligibility criteria in section 2.1, paragraph 1:

To be eligible to complete the survey participants had live in Canada and be aged 13- 18.

**A phrase is useful to concisely describe whether you use convenience or probability sampling and whether the stratification aims to ensure national representativeness.
A response rate should be provided. Non-responses should be characterized and compared to responses in terms of sociodemographic characteristics to address response and sample selection biases.**
We utilized quota sampling from a panel study, which are both are non-probability sampling approaches. Here the quotas were to allow a diversity of perspectives rather than based on population proportions. Although the underlying online panel aims to be nationally representative (https://www.qualtrics.com/research-services/online-sample/), we cannot state that our sample is, as it utilizes a quota approach drawn from a specific sub-group (13-18 year olds). We therefore use “quota sampling drawn from an online panel” to describe our approach, and we have added that we cannot make claims regarding the sample being representative of the population- we now clearly state this in the limitations (Limitations Section, Paragraph 1). We have also stated in the methods that this a “non-probability sample” (Section 2.1, Paragraph 2) and provide a citation that clearly outlines quota and online research panels as non-probability samples (<https://www150.statcan.gc.ca/n1/edu/power-pouvoir/ch13/nonprob/5214898-eng.htm>). We also note that the goal of qualitative research is to provide an in-depth, richer understanding of a phenomenon, rather than generalizability.

Further, using a research panel means we are only have access to sociodemographic data on participants who wish to take part in the study as they are invited to participate through the panel company by email and if they choose to not access the study, or simply do not open the email regarding it, we don’t have access to their information. We do note that 13,884 invitations were sent; however, this was done in a phased way given the quota sampling approach with some populations i.e., the younger group and those in the Territories being harder to reach for the quota; further, once a quota was met those individuals were no longer targeted by the panel company, so calculating a response rate from this value would be misleading. We can say that from those who clicked the link and were directed to the LOI (n=1542); 87.22% (n=1345) agreed to participate in the study, but we do not have any demographic data on these participants for comparison as they were not directed to the survey if they did not consent to participate. From there (895 met the eligibility check screening questions); Qualtrics, the panel company, provided initial quality checks for things such as straight lining, click throughs, or duplicate responses; which left us with 809 participants from the panel; from there we did a further data quality check by checking for strainghtlining and observing open text responses, and were left with a sample of 804. We now summarize this in the Methods Section in paragraph 3.

**3) Measure: You seem to have used different tenses interchangeably throughout the paper. Your question used present tense “is impacting.” In alignment with that, you should use “was impacting” rather than “impacted” or “impact.” This is not merely language policing, but this focus on the “present” should somehow reflect in your theorization of the constructs and your interpretation of the findings.**

We have revised the tense consistency throughout.

**4) Limitations: The open-ended responses may still suffer from common measurement errors of self-reports, including social desirability and recall biases, which should be acknowledged as a limitation.**

We have added potential for social desirability and recall bias as limitations of the study.
